# Retinoic Acid-Induced 1 Gene and Neuropsychiatric Diseases: A Systematic Review

**DOI:** 10.1017/erm.2025.12

**Published:** 2025-05-29

**Authors:** Tianmi Yang, Dejiang Pang, Chunyu Li, Huifang Shang

**Affiliations:** 1 Department of Neurology, West China Hospital, Sichuan University, Sichuan, China; 2 Laboratory of Neurodegenerative Disorders, West China Hospital, Sichuan University, Sichuan, China; 3 National Clinical Research Center for Geriatrics, West China Hospital, Sichuan University, Sichuan, China

**Keywords:** neuropsychiatric diseases, Potocki–Lupski syndrome, *RAI1*, retinoic acid-induced 1, Smith–Magenis syndrome, systematic review

## Abstract

**Background:**

Retinoic acid-induced 1 (*RAI1*) is a dosage-sensitive gene implicated in a range of rare neuropsychiatric diseases.

**Methods:**

This review provides a comprehensive overview of *RAI1’s* role, integrating both clinical and basic research on Smith–Magenis syndrome (SMS) and Potocki–Lupski syndrome (PTLS) while also summarising research progress on its involvement in spinocerebellar ataxia (SCA), autism spectrum disorder (ASD), schizophrenia, bipolar disorder and major depression. A systematic review of the literature was conducted using PubMed and EMBASE, following the PRISMA guidelines, with the protocol registered in PROSPERO (CRD42023474165).

**Results:**

A total of 99 eligible studies on *RAI1* were included. We presented detailed characterisations of SMS and PTLS patients, emphasising the crucial role of *RAI1* haploinsufficiency and overexpression in their pathogenesis. Additionally, we summarised research progress on *RAI1* in SCA, ASD, schizophrenia, bipolar disorder and major depression. Integrating findings from animal studies, particularly those examining the regulatory mechanisms of *RAI1* in critical phenotypes, such as body weight, sleep and epilepsy, underscores the precise regulation of *RAI1* expression in maintaining various nervous system functions.

**Conclusions:**

Overall, this review contributes to the identification of *RAI1*-related neuropsychiatric diseases, with a particular emphasis on enhancing clinical diagnosis of SMS and PTLS in developing countries.

## Introduction

The retinoic acid-induced 1 (*RAI1*) gene, located on chromosome 17p11.2, encodes a nuclear protein that plays a crucial role in maintaining normal neuropsychic functions (Ref. 1). Reviewing the research history of *RAI1* reveals its intricate association with two rare neurodevelopmental syndromes – Smith–Magenis syndrome (SMS) (OMIM: 182290) and Potocki–Lupski syndrome (PTLS) (OMIM: 610883) – which share overlapping features while also exhibiting distinct, syndrome-specific phenotypes (Refs 2, 3). It has long been recognised that *RAI1* is situated within the crucial gene region linked to both SMS and PTLS (Refs 4, 5). Until 2003, Slager et al. (Ref. 6) identified frameshift mutations in *RAI1* among SMS patients, confirming the central role of *RAI1* in this syndrome.

The clinical manifestations of SMS and PTLS in paediatric patients are complex, encompassing a broad spectrum of neurological, psychiatric and behavioural abnormalities (Refs 5, 7). As the Human Genome Project progresses, *RAI1* has been gradually recognised as being associated with various neuropsychiatric diseases. For instance, expansion of cytosine–adenine–guanine (CAG) repeats in *RAI1* has been implicated in modifying spinocerebellar ataxia (SCA) (Ref. 8). Large-scale genetic analyses in autism spectrum disorder (ASD) cohorts have also pinpointed *RAI1* as a susceptibility gene for ASD (Ref. 9). Furthermore, postmortem analyses of individuals with schizophrenia, bipolar disorder or major depression have demonstrated significantly elevated *RAI1* expression in brain tissues (Ref. 10). Recent foundational research has delved into the potential pathogenic mechanisms of *RAI1*, offering further insights into its role in neuropsychiatric diseases (Refs 11, 12, 13, 14, 15).

Given the critical role of *RAI1* in nervous system function, we performed a systematic review summarising recent advances in both clinical and basic research on *RAI1* in SMS and PTLS while providing an overview of research progress on its involvement in SCA, ASD, schizophrenia, bipolar disorder and major depression. This review aims to enhance our understanding of *RAI1*’s involvement across these conditions, with a particular emphasis on promoting the early recognition and diagnosis of SMS and PTLS.

## Methods

### Search strategy

This systematic review followed the Preferred Reporting Items for Systematic Reviews and Meta-Analyses (PRISMA) guidelines (Ref. 16) (Supplementary Material 1). The final search was conducted in the PubMed and EMBASE databases on 1 October 2024. The search strategy used the terms ‘Retinoic acid-induced 1’ OR ‘*RAI1*’ in the ‘All Fields’ option to ensure comprehensive identification of relevant studies. Additionally, the reference lists of pertinent reviews and articles were manually searched to capture any potentially missed references.

The review protocol was registered in PROSPERO (CRD42023474165) and is available at https://www.crd.york.ac.uk/PROSPERO/#recordDetails.

### Study selection

Articles were considered eligible if they met the following criteria: (1) original research conducted in English, (2) reporting on *RAI1* in neuropsychiatric diseases, including SMS, PTLS, SCA, ASD, schizophrenia, bipolar disorder and major depression, (3) providing explicit details on genetic and phenotypic information for case reports and case series studies and (4) published before the last search date (1 October 2024). Two authors (T.Y. and D.P.) independently evaluated the titles, abstracts and full texts of the identified articles to determine eligibility. Any discrepancies were resolved through discussion or consultation with a third author if necessary.

### Data extraction and quality assessment

The included studies were categorised into clinical and basic research based on the research theme. Clinical studies were defined as those involving patient cohorts, genetic analyses or phenotypic characterisations, whereas basic research included studies investigating molecular mechanisms, cellular functions and animal models of *RAI1.* The quality of all clinical studies was assessed using the Joanna Briggs Institute Critical Appraisal Checklist, where applicable (Ref. 17). Since SMS and PTLS patients share overlapping neurological and psychiatric symptoms, a standardised extraction form was used to collect detailed genotype and phenotype information for each case. This included the first author’s name, publication year, data source (country), disease, study design, sample size, gene sequencing method, variation pattern, deletion/duplication size (Mb), fragment location, mutation site, experimental validation of *RAI1* transcription and expression changes, coexisting mutations, sex, age, clinical features and auxiliary examinations. Positive clinical phenotypes were recorded when explicitly stated in the original articles, whereas negative or unspecified phenotypes were treated as negative. Gene positions were standardised according to the human reference genome GRCh37 (hg19). For basic research studies, the focus was on investigating the effects of *RAI1* deletion or duplication in animal models, along with the regulatory networks upstream and downstream of *RAI1.*

### Statistics

Chi-square tests were performed to compare categorical clinical phenotype variables between SMS and PTLS patients. A two-sided *p*-value of <0.05 was considered statistically significant.

## Results

### Studies retrieved and characteristics

**Figure 1.**
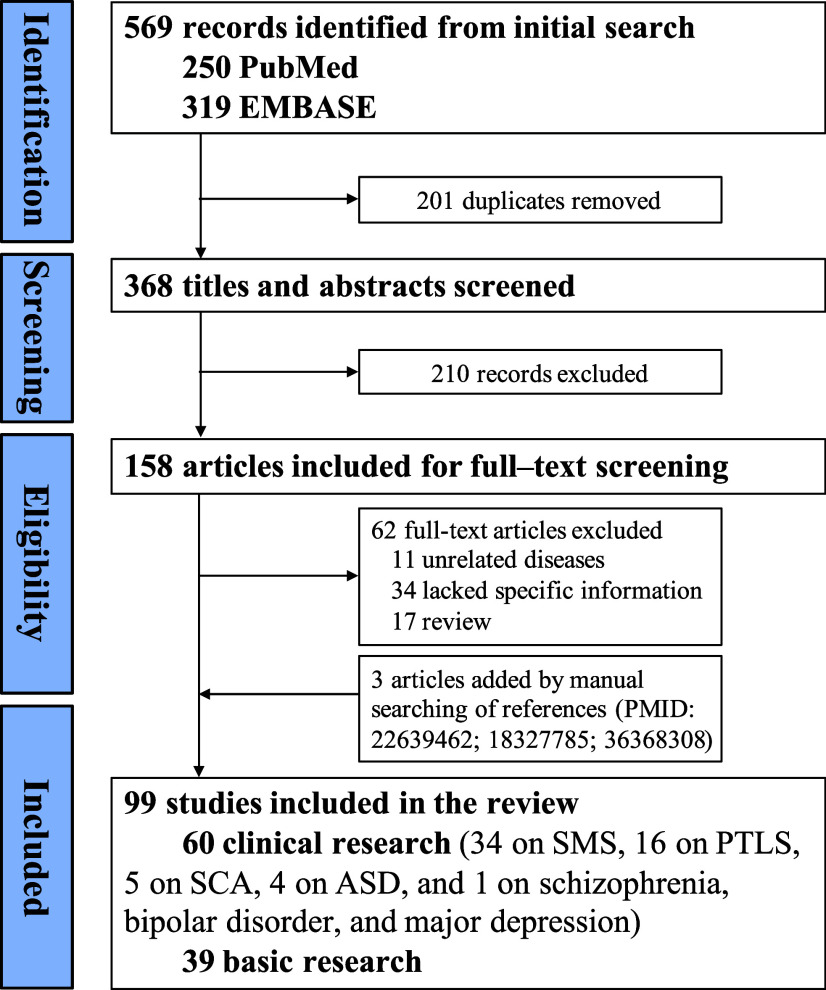
Flowchart of study selection. ASD: autism spectrum disorder; PTLS: Potocki–Lupski syndrome; SCA: spinocerebellar ataxia; SMS: Smith–Magenis syndrome.

Database searches identified 569 records. After removing duplicates (*n* = 201) and screening titles and abstracts (*n* = 210), 158 full-text articles were assessed. Ultimately, 99 articles met the eligibility criteria and were included (Figure 1). These comprised 60 clinical studies (34 on SMS, 16 on PTLS, 5 on SCA, 4 on ASD and 1 on schizophrenia, bipolar disorder and major depression), and 39 basic research studies. Full details of quality assessment for the included clinical studies were available in Supplementary Material 2.

A total of 83 SMS patients and 64 PTLS patients were included in this review, with no overlapping cases. Detailed variant and phenotypic information is provided in Supplementary Material 3. To date, this represents the largest and most comprehensive summary of SMS and PTLS cases. Notably, there is a marked geographic imbalance in case distribution, with approximately 51% (42/83) of SMS cases and 67% (43/64) of PTLS cases reported from the United States (Refs 5, 6, 18, 19, 20, 21, 22, 23,24, 25, 26, 27, 28, 29, 30, 31, 32, 33, 34, 35, 36, 37, 38, 39), and 24% (20/83) of SMS cases and 27% (17/64) of PTLS cases from Europe (Refs 40, 41, 42, 43, 44, 45, 46, 47, 48, 49, 50). In contrast, reports from Asia and Africa remain scarce. Thus, this work will improve awareness and facilitate the identification of *RAI1*-related diseases, particularly in underrepresented regions.

In basic research, mouse models for SMS and PTLS have successfully replicated many of the phenotypes observed in human patients (Refs 11, 51, 52, 53, 54, 55, 56). Preliminary studies have also explored potential therapeutic strategies for SMS and PTLS, identifying key therapeutic windows (Refs 13, 57, 58, 59) Notably, animal studies have provided valuable insights into the mechanisms underlying the complex phenotypes of these disorders, including abnormal body weight, sleep disturbances, circadian rhythm disruptions and seizures (Refs 12, 14, 55, 57, 58, 60, 61, 62, 63). The following section offers a comprehensive review and synthesis of these findings.

### Clinical research on RAI1 in neuropsychiatric diseases

#### RAI1 in Smith–Magenis syndrome and Potocki–Lupski syndrome

**Figure 2.**
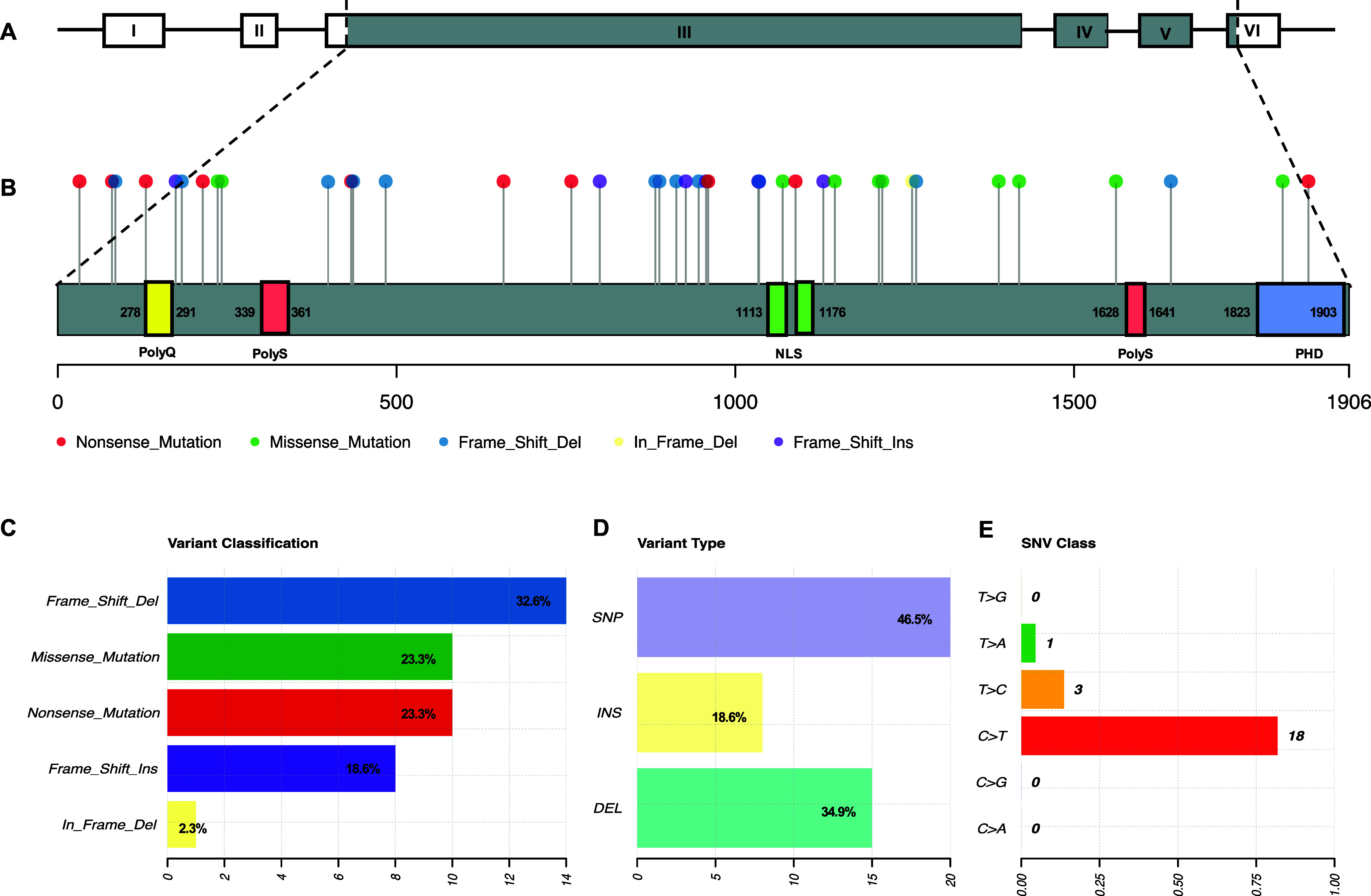
*RAI1* point mutations in Smith–Magenis syndrome. **A.** *RAI1* gene structure. **B.** *RAI1* protein structure with the distribution of point mutations. **C–E.** Classification of *RAI1* point mutations.

SMS and PTLS are rare neurodevelopmental disorders that present unique challenges in paediatric care. Both conditions are caused by alterations in the dosage of the *RAI1* gene (Refs 2, 3). SMS results from *RAI1* haploinsufficiency, typically due to 17p11.2 deletions (most commonly ~3.7 Mb) or pathogenic mutations, whereas PTLS is caused by *RAI1* overexpression resulting from 17p11.2 microduplications (0.25–11.2 Mb) (Supplementary Material 3). To date, 43 pathogenic *RAI1* point mutations have been identified in SMS patients (Supplementary Material 3). Among these, 51.2% are frameshift mutations, 23.3% are missense variants and 23.3% are nonsense mutations (Figure 2).

While SMS and PTLS share overlapping clinical features – such as craniofacial abnormalities, developmental delays, and behavioural challenges – distinct phenotypic patterns are evident (Refs 2, 3). Based on the types of variations involved in the *RAI1* gene, we summarised the clinical manifestations of SMS and PTLS patients in Table 1. Overall, SMS is often characterised by brachycephaly, midface hypoplasia, scoliosis, cognitive impairment, sleep disturbances, seizures, self-injurious behaviour and obesity. In contrast, PTLS is associated with micrognathia, slanting palpebral fissures, malformed ears, renal anomalies, underweight and muscle weakness.

**Table 1.** Phenotypic features in patients with SMS and PTLS

	SMS				PTLS	*p* ^b^
	Deletion (*n* = 38)	Point mutation (*n* = 45)	*p* ^a^	All SMS (*N* = 83)	Duplication (*N* = 64)	
*Physical developmental abnormalities*						
Brachycephaly/microcephaly	21 (55.26%)	22 (48.89%)	0.42	43 (51.81%)	17 (26.56%)	0.001
Midface hypoplasia	27 (71.05%)	27 (60.00%)	0.3	54 (65.06%)	16 (25.00%)	<0.001
Micrognathia	4 (10.53%)	3 (6.67%)	0.28	7 (8.43%)	20 (31.25%)	<0.001
Broad square face	23 (60.53%)	21 (46.67%)	0.13	44 (53.01%)	9 (14.06%)	<0.001
Deep-set/wide-set eyes	10 (26.32%)	7 (13.33%)	0.16	17 (20.48%)	4 (6.25%)	0.02
Strabismus/myopia/hyperopia/iris abnormalities/retinal detachment	16 (42.11%)	20 (44.44%)	0.66	36 (43.37%)	26 (40.63%)	0.82
Slanting palpebral fissures/palpebral ptosis	5 (13.16%)	8 (17.78%)	0.76	13 (15.66%)	24 (37.50%)	0.001
Synophrys/arched eyebrows	9 (23.68%)	9 (20.00%)	0.9	18 (21.69%)	4 (6.25%)	0.01
Lip/palate/epiglottis abnormality	11 (28.95%)	19 (42.22%)	0.3	30 (36.14%)	15 (23.44%)	0.08
Hoarse voice	12 (31.58%)	15 (33.33%)	0.88	27 (32.53%)	0	<0.001
Dental anomalies	14 (36.84%)	11 (24.44%)	0.21	25 (30.12%)	12 (18.75%)	0.07
Hearing loss	14 (36.84%)	11 (24.44%)	0.21	25 (30.12%)	4 (6.25%)	<0.001
Chronic ear infections	11 (28.95%)	19 (42.22%)	0.21	30 (36.14%)	1 (1.56%)	<0.001
Malformed ears	4 (10.53%)	4 (8.89%)	0.52	8 (9.64%)	17 (26.56%)	0.006
Short stature	20 (52.63%)	15 (33.33%)	0.07	35 (42.17%)	15 (23.44%)	0.02
Scoliosis/vertebral abnormalities	13 (34.21%)	12 (26.67%)	0.5	25 (30.12%)	5 (7.81%)	0.002
Brachydactyly/syndactyly/camptodactyly	23 (60.53%)	27 (60.00%)	0.97	50 (60.24%)	10 (15.63%)	<0.001
Foot deformities	5 (13.16%)	8 (17.78%)	0.77	13 (15.66%)	17 (26.56%)	0.08
*Neurological manifestations*						
Cognitive impairment	33 (86.84%)	34 (75.56%)	0.2	67 (80.72%)	39 (60.94%)	0.009
Speech delay	34 (89.47%)	37 (82.22%)	0.35	71 (85.54%)	59 (92.19%)	0.20
Motor delay	30 (78.95%)	34 (75.56%)	0.72	64 (77.11%)	48 (75.00%)	0.79
Sleep disturbance	33 (86.84%)	44 (97.78%)	0.05	77 (92.77%)	25 (39.06%)	<0.001
Seizures	11 (28.95%)	12 (26.67%)	0.81	23 (27.71%)	10 (15.63%)	0.04
Hypotonia	24 (63.16%)	22 (48.89%)	0.12	46 (55.42%)	44 (68.75%)	0.08
Muscle weakness	1 (2.63%)	0	NA	1 (1.20%)	13 (20.31%)	<0.001
Tendon hyperreflexia	6 (15.79%)	0	NA	6 (7.23%)	3 (4.69%)	0.51
Sensory loss	3 (7.89%)	8 (17.78%)	0.19	11 (13.25%)	4 (6.25%)	0.16
Headache	0	1 (2.22%)	NA	1 (1.20%)	1 (1.56%)	0.86
*Psychiatric/behavioural issues*						
Self-hugging	14 (36.84%)	19 (42.22%)	0.63	33 (39.76%)	0	<0.001
Self-injurious behaviour	30 (78.95%)	34 (75.56%)	0.86	64 (77.11%)	3 (4.69%)	<0.001
Aggressive behaviour	13 (34.21%)	28 (62.22%)	0.01	41 (49.40%)	8 (12.50%)	<0.001
Compulsive behaviour/motor stereotypes	6 (15.79%)	21 (46.67%)	0.001	27 (32.53%)	8 (12.50%)	0.007
Hyperactivity	10 (26.32%)	11 (24.44%)	0.87	21 (25.30%)	8 (12.70%)	0.1
Autistic features	5 (13.16%)	13 (28.89%)	0.04	18 (21.69%)	20 (31.70%)	0.14
Schizophrenia	0	1 (222%)	NA	1 (1.20%)	0	NA
Depression/anxiety	0	11 (24.44%)	NA	11 (13.25%)	8 (12.50%)	0.88
Overeating	2 (5.26%)	14 (31.11%)	0.003	16 (19.28%)	0	<0.001
*Other features*						
Overweight/obesity	8 (21.05%)	26 (57.78%)	0.001	34 (40.96%)	0	<0.001
Underweight/malnutrition	5 (13.16%)	1 (2.22%)	NA	6 (7.23%)	19 (20.21%)	<0.001
Cardiovascular defects	11 (26.95%)	7 (15.56%)	0.13	18 (21.69%)	14 (21.88%)	0.72
Cerebral venous thrombosis	1 (2.63%)	0	NA	1 (1.20%)	0	NA
Renal/urinary tract defect	1 (2.63%)	0	NA	1 (1.20%)	9 (14.06%)	0.003
Diaphragmatic hernia/inguinal hernia	1 (2.63%)	1 (2.22%)	0.9	2 (2.41%)	1 (1.56%)	0.71
Skin anomalies	1 (2.63%)	2 (4.44%)	NA	3 (3.61%)	0	NA

NA: not applicable; PTLS: Potocki–Lupski syndrome; SMS: Smith–Magenis syndrome.

a Comparison of phenotype between SMS patients with 17p11.2 deletion and RAl1 point mutation.

b Comparison of phenotype between all SMS patients and PTLS patients.

For paediatricians, recognising these differences is crucial for accurate diagnosis and tailored management. Of particular note, some phenotypes are syndrome-specific: overweight/obesity is observed only in SMS, whereas underweight/malnutrition and muscle weakness are primarily reported in PTLS. These findings emphasise the nuanced effects of *RAI1* gene dosage, which exhibit overlapping but distinct impacts on neurodevelopment. Understanding these variations can guide early diagnosis, intervention and long-term care, especially in resource-limited settings where SMS and PTLS may be underdiagnosed.

In patients with SMS, physical development and neurological features are largely comparable between individuals carrying 17p11.2 deletions and those with *RAI1* point mutations. However, patients with *RAI1* mutations exhibit a higher prevalence of sleep disturbances, aggressive behaviour, compulsive behaviour/motor stereotypes, autistic traits, overeating and overweight/obesity compared with those with 17p11.2 deletions (Table 1). Notably, larger deletions spanning approximately 5–10 Mb may encompass *RAI1*-adjacent genes, such as *PMP22*, leading to *PMP22-RAI1* deletions. Compared with the typical 3.7 Mb deletions seen in SMS, individuals with larger deletions are more likely to exhibit clinical signs of peripheral neuropathy and/or objective electrophysiological abnormalities (Supplementary Material 3), along with an earlier onset and increased severity of peripheral neuropathy (Ref. 36). These findings suggest that variability in 17p11.2 deletion sizes affects not only *RAI1* but also neighbouring genes, potentially contributing to distinct clinical phenotypes.

Most case studies have provided some clues regarding the variant patterns, *RAI1* expression levels and their relationship to the diagnosis of SMS and PTLS. Typically, *RAI1* deletions and nonsense mutations reduce gene expression and are linked to SMS, while duplications increase expression and lead to PTLS. However, exceptions, particularly involving missense mutations or regulatory region variants, challenge this framework. For instance, individuals carrying missense mutations in *RAI1* (e.g., p.Y236F, p.T1061T, p.Q1389R and p.R1217Q) exhibit SMS features, yet Western blotting or quantitative PCR often shows unchanged *RAI1* expression levels (Refs 28, 64). Similarly, a case with the p.R1147Q de novo mutation demonstrated a normal protein level and localisation but impaired transcriptional activation of brain-derived neurotrophic factor (*BDNF*), suggesting abnormal regulatory activity as a potential mechanism (Ref. 37).

PTLS cases also reveal complexity. One patient with no changes in *RAI1* copy number carried a maternally inherited upstream deletion that disrupted regulatory elements, leading to elevated *RAI1* mRNA levels (Ref. 32). Conversely, an SMS case with a deletion involving exons 5 and 6 paradoxically resulted in increased *RAI1* transcript levels due to loss of the canonical stop codon, replaced by a downstream alternative (Ref. 50). These unique cases underscore the nuanced relationship between *RAI1* variant types, expression levels and functional activity. They also highlight diagnostic challenges in distinguishing SMS from PTLS and suggest that clinical evaluation should consider both variant patterns and functional consequences of *RAI1* dysregulation.

#### RAI1 in spinocerebellar ataxia

SCA comprises a group of inherited disorders characterised by progressive degeneration of the cerebellum, brainstem and spinal cord (Ref. 65). Among its subtypes, SCA1 and SCA2 are caused by CAG repeat expansions in *ATXN1* and *ATXN2*, where repeat length correlates with earlier disease onset (Refs 65, 66). *RAI1* contains a polymorphic CAG repeat tract and has been implicated in polyglutamine disorders (Ref. 67). Studies suggest a potential role for *RAI1* in modifying the onset of SCA. In a study of 46 SCA2 patients, the repeat size of *RAI1* was found to explain an additional 4.1% of the variation in age of onset after accounting for the impact of ATXN2 expansions (Ref. 8). Similarly, research on 23 independent families with SCA2 in India supported this finding, suggesting that *RAI1* CAG repeat variation contributed to approximately 13% of the unexplained variation in age of onset for SCA2 (Ref. 68). However, these results were not validated in a South American SCA2 cohort, where no association was found between *RAI1* repeat size and disease onset or clinical phenotype (Ref. 69). In SCA1, a study involving 152 patients in China found no significant modifying effect of *RAI1* CAG repeats on disease onset (Ref. 70). While these studies provide preliminary evidence for a potential modifying role of *RAI1*, the overall link between *RAI1* and SCA remains inconclusive and requires further investigation with larger and more diverse cohorts.

#### RAI1 in autism spectrum disorder, schizophrenia, bipolar disorder and major depression

Genetic studies from the Netherlands and France have identified *RAI1* as a potential susceptibility gene for ASD (Refs 71, 72). Network analyses of gene interactions have further revealed connections between *RAI1* and multiple ASD-related proteins, suggesting their involvement in shared molecular pathways (Ref. 73). In the most recent and extensive global whole-genome sequencing study, which included 5,100 individuals with ASD and 6,212 non-ASD parents and siblings, identified *RAI1* as significantly associated with ASD (Ref. 9). Evidence for *RAI1*’s involvement in schizophrenia, bipolar disorder and major depression is relatively limited. A post-mortem study reported significantly increased *RAI1* expression in the dorsolateral prefrontal cortex of patients with these psychiatric disorders (Ref. 10). However, a genome-wide association study found no significant association between *RAI1* and schizophrenia risk (Ref. 74). These findings suggest that *RAI1* is crucial for mental health, not only during early development but potentially throughout the lifespan, warranting further research into its broader neuropsychiatric implications.

### Basic research on RAI1 in Smith–Magenis syndrome and Potocki–Lupski syndrome

Animal model studies have been instrumental in elucidating the essential role of *RAI1* in mammalian development and homeostasis, shedding light on its involvement in SMS and PTLS. Walz et al. (Ref. 51) engineered mouse models carrying either a deletion (Df(11)17) or duplication (Dp(11)17) in a chromosomal region syntenic to human 17p11.2, commonly affected in SMS and PTLS. Df(11)17/+ mice exhibited craniofacial abnormalities, seizures, significant obesity and male-specific reduced fertility, whereas Dp(11)17/+ mice were underweight and did not experience seizures (Ref. 51). No apparent histological defects were observed in the hearts, spleens or kidneys of either model (Ref. 51), highlighting the specific phenotypic consequences of these alterations. To minimise confounding effects from neighbouring genes in the 17p11.2 region, researchers developed transgenic mice with altered *Rai1* expression. The findings emphasised *Rai1* as a dosage-sensitive gene critical for central nervous system function. Homozygous *Rai1*^−/−^ mice exhibited severe phenotypes, with most dying during early development (Refs 53, 75). Rare survivors demonstrated growth retardation, craniofacial malformations, seizures, motor impairment and cognitive deficits (Refs 53, 75). In contrast, heterozygous *Rai1*^+/−^ mice exhibited SMS-like traits, including craniofacial anomalies, obesity, seizures, learning difficulties and social behaviour abnormalities (Refs 53, 75, 76).

Girirajan et al. (Ref. 54) graded overexpression of *Rai1*, finding that animals with 1.5- to 2-fold *Rai1* overexpression showed PTLS-like features, such as growth retardation, reduced body weight, increased anxiety-like behaviours and heightened locomotor activity. Additionally, in *Rai1* conditionally transgenic mice with *Rai1* specifically overexpressed in the hippocampus, cerebral cortex, striatum, amygdala and olfactory bulb, significant weight loss was also observed (Ref. 56).

Besides these phenotypic studies of animal models, the mechanisms underlying *Rai1* regulation of weight abnormalities, disrupted sleep rhythms and epileptic seizures have been investigated. Such studies provide foundational insights for understanding and potentially managing SMS and PTLS in clinical settings.

#### RAI1 and weight

*RAI1* haploinsufficiency has emerged as a monogenic model for obesity. Studies in *Rai1*^+/−^ mice reveal significant weight gain compared to wild-type (WT) mice, with weight differences apparent by 20 weeks of age under standard feeding conditions (Ref. 60). Both male and female *Rai1*^+/−^ mice exhibited sustained weight gain into adulthood, approximately doubling the weight gain rate of WT mice (Ref. 60). This was accompanied by hyperphagia, impaired satiety and increased abdominal and subcutaneous fat distribution (Ref. 60). Interestingly, metabolic syndrome was not initially observed in these mice, as levels of adiponectin, insulin, glucose, total cholesterol and triglycerides were comparable to WT controls (Ref. 60). However, later studies on *Rai1*^+/−^ mice reported metabolic alterations, suggesting that genetic background may influence metabolic phenotypes (Ref. 77). In contrast, Df(11)17/+ mice – carrying a broader chromosomal deletion – exhibited metabolic syndrome characteristics, including elevated fat content, reduced high-density lipoprotein levels and decreased insulin sensitivity (Ref. 55). This discrepancy suggests that neighbouring genes near *Rai1* may modulate metabolic phenotypes, complicating the relationship between *RAI1* and obesity (Refs 55, 60).

Mechanistic insights were provided by RNA sequencing of the hypothalamus in *Rai1*^+/−^ mice, which revealed reduced expression of *BDNF.* Chromatin immunoprecipitation (ChIP)-Chip and luciferase reporter assays demonstrated that *RAI1* regulates BDNF transcription via an intronic enhancer (Ref. 60). Javed et al. (Ref. 58) further advanced understanding through an inducible *Rai1* knockout mouse model, allowing targeted deletion of *Rai1* postnatally. Deleting *Rai1* at 3 or 8 weeks of age did not affect neurobehavioural functions but induced hyperphagia and obesity (Ref. 58). Notably, selective overexpression of *BDNF* – either murine or human – in the paraventricular or ventromedial hypothalamic nuclei successfully reversed weight gain and hyperphagia in *Rai1*^+/−^ mice (Ref. 58). Building on these findings, researchers applied paraventricular nucleus-specific CRISPR activation using recombinant adeno-associated virus during early adolescence (3–4 weeks of age) in *Rai1*^+/−^ mice (Ref. 59). This therapy boosted endogenous *Rai1* expression, delayed obesity onset and partially alleviated hyperphagia (Ref. 59). Javed et al. (Ref. 77) further refined our understanding of *RAI1*’s role in regulating body weight, revealing its impact on hypothalamic *BDNF*-producing neurons, particularly those in the paraventricular nucleus. The study demonstrated that loss of *Rai1* from these *BDNF*-producing neurons induces obesity in mice, with alterations in neuronal excitability and energy homeostasis (Ref. 77). Interestingly, the researchers identified LM22A-4, a small molecule that activates neurotrophin signalling, which delayed obesity onset and partially improved metabolic phenotypes in *Rai1*+/− mice (Ref. 77).

Evidences above underscore the pivotal role of *RAI1* in hypothalamic energy balance regulation via postnatal *BDNF* expression. Early interventions targeting *RAI1*, particularly before adolescence, hold promise for mitigating SMS-related obesity and associated behavioural phenotypes (Refs 13, 59, 58).

#### RAI1 and sleep disturbance

Sleep disturbances are a hallmark of SMS and PTLS, but they are significantly more prevalent in SMS patients (92.8%) compared with PTLS patients (39.1%; *p* < 0.001) (Table 1). Both patient data and animal models highlight the critical role of *RAI1* in circadian rhythm regulation, with *RAI1* haploinsufficiency appearing more disruptive than overexpression. Animal studies reveal that Df(11)17/+ mice and *Rai1*^+/−^ mice exhibit marked circadian abnormalities, primarily characterised by a shortened period length (Refs 52, 61, 62, 63). In contrast, Dp(11)17/+ mice showed either normal or milder circadian rhythm disturbances (Refs 12, 52, 62). These observations support a dose-dependent effect of *RAI1* on circadian behaviour.

Mechanistic insights come from studies showing that *RAI1* knockdown alters the expression of key circadian components, including *CLOCK* and other critical genes in the circadian feedback loop (Ref. 61). This disruption has been observed in human cell lines (HEK 293T, U2OS-B), fibroblasts derived from SMS patients and the hypothalamus of *Rai1*^+/−^ mice (Ref. 61). ChIP-Chip and luciferase data confirmed *RAI1* as a positive regulator of *CLOCK* and an integral part of the circadian transcription loop (Ref. 61). Interestingly, Dp(11)17/+ mice also exhibit dysregulation of circadian genes (*CLOCK*, *Arntl*, *Per1–3* and *Cry1/2*) in the hypothalamus, indicating that *RAI1* overexpression can also impair circadian function (Ref. 12). This suggests that both insufficient and excessive *RAI1* disrupt the molecular clock’s transcriptional regulation. In addition to downregulating *CLOCK* expression, Diessler et al. (Ref. 63) highlighted the involvement of *Rai1* in processing non-visual light information, noting that *Rai1* haploinsufficiency increased the sensitivity of *Rai1*^+/−^ mice to light stimuli. This discovery offered a new mechanism to understand sleep disturbances in SMS patients.

The cumulative evidence supports the core role of *Rai1* in the central pathways of the sleep-wake cycle and circadian rhythm, indicating that both *Rai1* deficiency and overexpression disrupt the transcriptional regulation of the molecular clock.

#### RAI1 and seizures

Seizures or abnormal electroencephalogram patterns were observed in a subset of SMS patients (27.7%). The work of Chang et al. (Ref. 14) provided insights into the underlying mechanisms, indicating that the absence of *Rai1* enhanced hippocampal excitability and promoted epileptogenesis in SMS models. Specifically, the researchers employed whole-brain *Rai1* homozygous knockout mice, combined with in vivo and ex vivo electrophysiological recordings, as well as in vivo brain structural and metabolic imaging, to uncover the involvement of *Rai1* in modulating neuronal excitability (Ref. 14). In this context, hippocampal dentate gyrus granule cells exhibited heightened intrinsic excitability and augmented glutamatergic synaptic transmission (Ref. 14). In addition, insights from Garay et al. (Ref. 78) shed light on the synaptic *Rai1*-mediated gene transcription regulation, emphasising the unique role of *Rai1* in homeostatic synaptic plasticity. The reduced expression of *Rai1* induced functional changes in excitatory synaptic function, potentially contributing to alterations in neuronal excitability and the occurrence of seizures.

## Discussion

This review provides a systematic examination of *RAI1* and its involvement in a range of neuropsychiatric diseases, with particular relevance to paediatric conditions. In SMS and PTLS, *RAI1* mutations lead to clear neurological and behavioural challenges, including developmental delays, sleep disturbances and cognitive impairments. These findings underscore *RAI1*’s critical involvement in early brain development, which is especially important for paediatric healthcare providers. While some conditions, such as ASD, typically manifest in early childhood, others, such as schizophrenia and bipolar disorder, often emerge in late adolescence or early adulthood. In contrast, SCA is generally classified as a group of adult-onset neurodegenerative disorders. The involvement of *RAI1* in these conditions underscores its crucial role in maintaining neuropsychiatric health across different life stages.

Both reduced and overexpressed *RAI1* can lead to overlapping neuropsychiatric phenotypes. However, as mentioned above, the occurrence and severity of these overlapping symptoms differ between SMS and PTLS patients. The summarised evidences from rare case reports emphasise the dose sensitivity of *RAI1* during early embryonic development, highlighting the necessity for precise control of *RAI1* dosage throughout development to ensure appropriate neurobehavioural functions in adulthood. Although recent studies have linked *RAI1* to amyotrophic lateral sclerosis (Ref. 79) and Parkinson’s disease (Ref. 80), as well as benign adult familial myoclonic epilepsy type 8 (Ref. 81), its role in the mature nervous system remains poorly understood. Notably, current research has primarily focused on *RAI1’s* dosage sensitivity – that is, the effects of insufficient or excessive expression – while largely overlooking its intrinsic biochemical properties. For instance, nonsense and frameshift mutations occurring upstream of the nuclear localisation signal result in truncated proteins, which not only lead to *RAI1* haploinsufficiency but also raise the question of whether the mislocalised N-terminal fragment, retained in the cytoplasm, exerts dominant-negative or toxic effects. Furthermore, given that *RAI1* exceeds 200 kDa in molecular weight and contains intrinsically disordered regions, it may exhibit a propensity for aggregation. This raises a critical yet largely unexplored question: Does *RAI1* duplication not only increase expression but also promote aberrant self-aggregation? If so, what are the functional and pathological consequences of such aggregation? Addressing these fundamental questions will be essential for advancing our understanding of *RAI1’s* molecular properties and its broader implications in neurodevelopmental and neurodegenerative disorders.

In addition to chromosomal structural variations and point mutations, the *RAI1* gene is also susceptible to CAG repeat expansions (Ref. 1). Normal *RAI1* CAG repeats range from 10 to 19 in the general population, with 13 or 14 repeats being most common (Refs 22, 82, 83). Indeed, expansion of CAG repeats is associated not only with SCA and Huntington’s disease but also with other neurodegenerative conditions (Ref. 84). The finding that CAG repeat length in *RAI1* modifies the age of onset in SCA patients aligns with the broader theory that (CAG)*n* repeat expansions influence disease onset (Ref. 66). However, these findings need validation through larger and more diverse population studies, and further research is necessary to confirm the impacts at the cellular level.

Evidence from genome-wide association studies (Ref. 9) and post-mortem brain analyses (Ref. 10) strongly implicates *RAI1* in various psychiatric disorders, although direct research on its role in ASD, schizophrenia, bipolar disorder and major depression remains limited. Moreover, both SMS and PTLS patients present with significant psychiatric symptoms, such as autistic features, schizophrenia, compulsive behaviour, aggressive behaviour, self-injurious behaviour, depression and anxiety, suggesting that both reduced and elevated *RAI1* expression contribute to these phenotypes. In SMS and PTLS, these symptoms primarily stem from aberrant *RAI1* expression, whereas in other psychiatric disorders, *RAI1* likely acts within broader genetic networks influencing disease susceptibility and clinical presentation. Of course, further studies are needed to clarify the specific and shared roles of *RAI1* in psychiatric conditions and the underlying mechanisms.

*RAI1* is a highly conserved dosage-sensitive gene across species. Most fundamental studies have primarily focused on mouse models with altered gene expression to mimic SMS and PTLS, providing key mechanistic insights. However, there is currently no available evidence from other model organisms, such as zebrafish or Drosophila, highlighting the need for further investigation. As a transcription factor, *RAI1* regulates diverse biological processes, including neuronal differentiation, cell growth, skeletal development, lipid biosynthesis, glucose metabolism, behaviour and circadian rhythms (Ref. 85). In SMS, *RAI1* haploinsufficiency in the hypothalamus reduces *BDNF* and *CLOCK* expression, contributing to obesity and sleep disturbances, respectively (Refs 12, 58). Additionally, studies have linked *Rai1* deficiency in hippocampal and glutamatergic neurons to heightened excitability and enhanced excitatory synaptic transmission, underlying epilepsy phenotypes (Ref. 14). Mouse models have proven particularly effective for studying weight abnormalities, sleep disturbances and epilepsy, as these phenotypes are relatively easy to replicate and monitor. These investigations underscore the critical role of precisely regulated *RAI1* expression and transcriptional activity in maintaining normal brain function. Moreover, recent studies have further characterised the neuroanatomical distribution and molecular interactions of *RAI1* in both mice and primates (marmosets) (Ref. 86). Overall, the specific mechanisms by which *RAI1* contributes to a broader spectrum of neurological and psychiatric disorders require further exploration.

Several limitations of this study should be acknowledged. First, our review only included studies in the English language published in the PubMed and EMBASE databases, potentially missing some relevant published studies. Second, in the clinical research review section, the majority of reported SMS and PTLS patients were from the United States and European countries, limiting the representativeness of the samples and possibly not accounting for potential genetic and phenotypic differences in patients from other regions. Future studies with larger and more diverse cohorts are necessary to systematically quantify *RAI1* genotype–phenotype associations. Third, as illustrated in Table 1, SMS and PTLS patients manifested a diverse array of phenotypes, including but not limited to craniofacial and skeletal development, cognition, motor skills, sensory functions, psychiatric behaviour, cardiovascular system and urinary system development. However, in the basic research review section, we primarily focused on summarising the currently more extensive and in-depth mechanistic studies, specifically those related to the mechanisms of *RAI1* in regulating weight, circadian rhythm and epilepsy.

## Conclusions

This review highlights the role of *RAI1* in a range of neuropsychiatric diseases, including SMS, PTLS, SCA, ASD, schizophrenia, bipolar disorder and major depression. Starting with the summary of SMS and PTLS cases and their corresponding mouse model phenotypic mechanisms, we gained insights into the pivotal role of *RAI1* in neurodevelopment and functional maintenance, emphasising the importance of precise gene dosage. Future research endeavours should address several key aspects: (1) further exploring the mechanisms behind the diverse phenotypes observed in SMS and PTLS, (2) validating *RAI1*’s role in various neurological and psychiatric conditions and (3) expanding our understanding of its regulatory network in the adult brain. A deeper understanding of *RAI1*’s functions will not only enhance knowledge of these disorders but also pave the way for potential therapeutic interventions.

## Supporting information

Yang et al. supplementary material 1 Yang et al. supplementary material

Yang et al. supplementary material 2 Yang et al. supplementary material

## Data Availability

All data generated or analysed during this study are included in this published article (and its Supplementary Materials).

## Supplementary material

The supplementary material for this article can be found at http://doi.org/10.1017/erm.2025.12.
