# Supplementary material for: Retinoic Acid-Induced 1 Gene and Neuropsychiatric Diseases: A Systematic Review
Source: Expert Rev Mol Med. 2025 May 29;27:e17. doi: 10.1017/erm.2025.12 (PMC12133160; doi:10.1017/erm.2025.12)
Supplement: Yang et al. supplementary material 1 — Yang et al. supplementary material [file S1462399425000122sup001.docx]

**Additional file 1. PRISMA 2020 Checklist (1)**

| **Section and Topic** | **Item #** | **Checklist item** | **Location where item is reported** |
| --- | --- | --- | --- |
| **TITLE** | | |  |
| Title | 1 | Identify the report as a systematic review. | Page 1 |
| **ABSTRACT** | | |  |
| Abstract | 2 | See the PRISMA 2020 for Abstracts checklist. | Page 2 |
| **INTRODUCTION** | | |  |
| Rationale | 3 | Describe the rationale for the review in the context of existing knowledge. | Page 3-4 |
| Objectives | 4 | Provide an explicit statement of the objective(s) or question(s) the review addresses. | Page 4 |
| **METHODS** | | |  |
| Eligibility criteria | 5 | Specify the inclusion and exclusion criteria for the review and how studies were grouped for the syntheses. | Page 4-5 |
| Information sources | 6 | Specify all databases, registers, websites, organisations, reference lists and other sources searched or consulted to identify studies. Specify the date when each source was last searched or consulted. | Page 4-5 |
| Search strategy | 7 | Present the full search strategies for all databases, registers and websites, including any filters and limits used. | Page 4 |
| Selection process | 8 | Specify the methods used to decide whether a study met the inclusion criteria of the review, including how many reviewers screened each record and each report retrieved, whether they worked independently, and if applicable, details of automation tools used in the process. | Page 4-5 |
| Data collection process | 9 | Specify the methods used to collect data from reports, including how many reviewers collected data from each report, whether they worked independently, any processes for obtaining or confirming data from study investigators, and if applicable, details of automation tools used in the process. | Page 5 |
| Data items | 10a | List and define all outcomes for which data were sought. Specify whether all results that were compatible with each outcome domain in each study were sought (e.g. for all measures, time points, analyses), and if not, the methods used to decide which results to collect. | Page 5 |
|  | 10b | List and define all other variables for which data were sought (e.g. participant and intervention characteristics, funding sources). Describe any assumptions made about any missing or unclear information. | Page 5 |
| Study risk of bias assessment | 11 | Specify the methods used to assess risk of bias in the included studies, including details of the tool(s) used, how many reviewers assessed each study and whether they worked independently, and if applicable, details of automation tools used in the process. | Page 5 |
| Effect measures | 12 | Specify for each outcome the effect measure(s) (e.g. risk ratio, mean difference) used in the synthesis or presentation of results. | NA |
| Synthesis methods | 13a | Describe the processes used to decide which studies were eligible for each synthesis (e.g. tabulating the study intervention characteristics and comparing against the planned groups for each synthesis (item #5)). | Additional file 3 |
|  | 13b | Describe any methods required to prepare the data for presentation or synthesis, such as handling of missing summary statistics, or data conversions. | Additional file 3 |
|  | 13c | Describe any methods used to tabulate or visually display results of individual studies and syntheses. | Page 6 |
|  | 13d | Describe any methods used to synthesize results and provide a rationale for the choice(s). If meta-analysis was performed, describe the model(s), method(s) to identify the presence and extent of statistical heterogeneity, and software package(s) used. | Page 6 |
|  | 13e | Describe any methods used to explore possible causes of heterogeneity among study results (e.g. subgroup analysis, meta-regression). | NA |
|  | 13f | Describe any sensitivity analyses conducted to assess robustness of the synthesized results. | NA |
| Reporting bias assessment | 14 | Describe any methods used to assess risk of bias due to missing results in a synthesis (arising from reporting biases). | NA |
| Certainty assessment | 15 | Describe any methods used to assess certainty (or confidence) in the body of evidence for an outcome. | NA |
| **RESULTS** | | |  |
| Study selection | 16a | Describe the results of the search and selection process, from the number of records identified in the search to the number of studies included in the review, ideally using a flow diagram. | Page 6 and Figure 1 |
|  | 16b | Cite studies that might appear to meet the inclusion criteria, but which were excluded, and explain why they were excluded. | NA |
| Study characteristics | 17 | Cite each included study and present its characteristics. | Page 6-16 and Additional file 3 |
| Risk of bias in studies | 18 | Present assessments of risk of bias for each included study. | NA |
| Results of individual studies | 19 | For all outcomes, present, for each study: (a) summary statistics for each group (where appropriate) and (b) an effect estimate and its precision (e.g. confidence/credible interval), ideally using structured tables or plots. | Table 1 and Additional file 3 |
| Results of syntheses | 20a | For each synthesis, briefly summarise the characteristics and risk of bias among contributing studies. | NA |
|  | 20b | Present results of all statistical syntheses conducted. If meta-analysis was done, present for each the summary estimate and its precision (e.g. confidence/credible interval) and measures of statistical heterogeneity. If comparing groups, describe the direction of the effect. | Table 1 and page 6-12 |
|  | 20c | Present results of all investigations of possible causes of heterogeneity among study results. | Page 6-7 |
|  | 20d | Present results of all sensitivity analyses conducted to assess the robustness of the synthesized results. | NA |
| Reporting biases | 21 | Present assessments of risk of bias due to missing results (arising from reporting biases) for each synthesis assessed. | NA |
| Certainty of evidence | 22 | Present assessments of certainty (or confidence) in the body of evidence for each outcome assessed. | NA |
| **DISCUSSION** | | |  |
| Discussion | 23a | Provide a general interpretation of the results in the context of other evidence. | Page 17-19 |
|  | 23b | Discuss any limitations of the evidence included in the review. | Page 19-20 |
|  | 23c | Discuss any limitations of the review processes used. | Page 19-20 |
|  | 23d | Discuss implications of the results for practice, policy, and future research. | Page 20-21 |
| **OTHER INFORMATION** | | |  |
| Registration and protocol | 24a | Provide registration information for the review, including register name and registration number, or state that the review was not registered. | Page 4 |
|  | 24b | Indicate where the review protocol can be accessed, or state that a protocol was not prepared. | Page 4 |
|  | 24c | Describe and explain any amendments to information provided at registration or in the protocol. | NA |
| Support | 25 | Describe sources of financial or non-financial support for the review, and the role of the funders or sponsors in the review. | Page 21 |
| Competing interests | 26 | Declare any competing interests of review authors. | Page 21 |
| Availability of data, code and other materials | 27 | Report which of the following are publicly available and where they can be found: template data collection forms; data extracted from included studies; data used for all analyses; analytic code; any other materials used in the review. | Additional file 3 |

**Additional file 2. Critical appraisal of clinical studies included in the systematic review**

| **Diseases** | **Ref.** | **First author** | **Year** | **Checklist criteria (2)** | | | | | | | | | | |
| --- | --- | --- | --- | --- | --- | --- | --- | --- | --- | --- | --- | --- | --- | --- |
|  |  |  |  | Q1 | Q2 | Q3 | Q4 | Q5 | Q6 | Q7 | Q8 | Q9 | Q10 | Total (%) |
| SMS | (3) | Stern et al. | 2024 | 2 | 2 | 2 | 2 | 0 | 2 | 1 | 2 |  |  | 81.25% |
| SMS | (4) | Cuk et al. | 2024 | 2 | 2 | 2 | 2 | 0 | 2 | 1 | 2 |  |  | 81.25% |
| SMS | (5) | Wu et al. | 2023 | 2 | 2 | 2 | 2 | 0 | 0 | 1 | 2 |  |  | 68.75% |
| SMS | (6) | Yu et al. | 2023 | 2 | 2 | 2 | 2 | 0 | 2 | 1 | 2 |  |  | 81.25% |
| SMS | (7) | Sironi et al. | 2022 | 2 | 2 | 2 | 2 | 0 | 2 | 1 | 2 |  |  | 81.25% |
| SMS | (8) | Onesimo et al. | 2022 | 2 | 2 | 2 | 2 | 1 | 2 | 1 | 2 |  |  | 87.50% |
| SMS | (9) | Kuroda et al. | 2022 | 2 | 2 | 2 | 2 | 0 | 2 | 0 | 2 |  |  | 75.00% |
| SMS | (10) | Akkus et al. | 2020 | 2 | 2 | 2 | 0 | 0 | 2 | 2 | 2 | 0 | 1 | 65.00% |
| SMS | (11) | Abad et al. | 2018 | 2 | 2 | 2 | 2 | 1 | 2 | 1 | 2 |  |  | 87.50% |
| SMS | (12) | Acquaviva et al. | 2017 | 2 | 2 | 2 | 2 | 1 | 2 | 0 | 2 |  |  | 81.25% |
| SMS | (13) | Yuan et al. | 2016 | 2 | 2 | 2 | 0 | 0 | 2 | 2 | 1 | 0 | 2 | 65.00% |
| SMS | (14) | Yeetong et al. | 2016 | 2 | 2 | 2 | 2 | 1 | 2 | 0 | 2 |  |  | 81.25% |
| SMS | (15) | Nijim et al. | 2016 | 2 | 2 | 2 | 2 | 2 | 2 | 1 | 2 |  |  | 93.75% |
| SMS | (16) | Thaker et al. | 2015 | 2 | 2 | 2 | 2 | 1 | 2 | 1 | 2 |  |  | 87.50% |
| SMS | (17) | Li et al. | 2015 | 2 | 2 | 2 | 2 | 0 | 2 | 2 | 2 |  |  | 87.50% |
| SMS | (18) | Dubourg et al. | 2014 | 2 | 2 | 2 | 0 | 0 | 2 | 2 | 1 | 0 | 2 | 65.00% |
| SMS | (19) | Capra et al. | 2014 | 2 | 2 | 2 | 2 | 0 | 2 | 1 | 2 |  |  | 81.25% |
| SMS | (20) | Adams et al. | 2014 | 2 | 2 | 2 | 1 | 0 | 2 | 1 | 2 |  |  | 75.00% |
| SMS | (21) | Vieira et al. | 2012 | 2 | 2 | 2 | 0 | 1 | 2 | 2 | 1 | 0 | 1 | 65.00% |
| SMS | (22) | Lee et al. | 2012 | 2 | 2 | 2 | 1 | 1 | 2 | 2 | 1 | 0 | 1 | 70.00% |
| SMS | (23) | Huang et al. | 2012 | 2 | 2 | 2 | 2 | 2 | 2 | 0 | 2 |  |  | 87.50% |
| SMS | (24) | Vilboux et al. | 2011 | 2 | 2 | 2 | 2 | 1 | 2 | 2 | 1 | 0 | 1 | 75.00% |
| SMS | (25) | Sanford et al. | 2011 | 2 | 2 | 2 | 2 | 0 | 1 | 1 | 2 |  |  | 75.00% |
| SMS | (26) | Gamba et al. | 2011 | 2 | 2 | 2 | 2 | 2 | 2 | 2 | 1 | 1 | 2 | 90.00% |
| SMS | (27) | Truong et al. | 2010 | 2 | 2 | 2 | 2 | 1 | 2 | 2 | 1 | 0 | 0 | 70.00% |
| SMS | (28) | Girirajan et al. | 2007 | 2 | 2 | 2 | 1 | 0 | 1 | 0 | 2 |  |  | 62.50% |
| SMS | (29) | Bi et al. | 2006 | 2 | 2 | 2 | 2 | 0 | 1 | 1 | 1 | 0 | 0 | 55.00% |
| SMS | (30) | Vlangos et al. | 2005 | 2 | 2 | 2 | 2 | 0 | 2 | 1 | 1 | 0 | 0 | 60.00% |
| SMS | (31) | Schoumans et al. | 2005 | 2 | 2 | 2 | 2 | 0 | 2 | 0 | 1 | 0 | 0 | 55.00% |
| SMS | (32) | Girirajan et al. | 2005 | 2 | 2 | 2 | 2 | 0 | 2 | 1 | 1 | 0 | 2 | 70.00% |
| SMS | (33) | Spadoni et al. | 2004 | 2 | 2 | 2 | 2 | 2 | 2 | 0 | 2 |  |  | 87.50% |
| SMS | (34) | Myers et al. | 2004 | 2 | 2 | 2 | 1 | 0 | 1 | 1 | 1 | 0 | 2 | 60.00% |
| SMS | (35) | Bi et al. | 2004 | 2 | 2 | 2 | 2 | 0 | 2 | 0 | 1 | 0 | 2 | 65.00% |
| SMS | (36) | Slager et al. | 2003 | 2 | 2 | 2 | 2 | 2 | 2 | 0 | 2 |  |  | 87.50% |
| PTLS | (37) | Deginet et al. | 2024 | 2 | 2 | 2 | 2 | 0 | 2 | 1 | 2 |  |  | 81.25% |
| PTLS | (38) | Grama et al. | 2021 | 2 | 2 | 2 | 2 | 0 | 2 | 2 | 2 | 0 | 1 | 75.00% |
| PTLS | (39) | Ciaccio et al. | 2020 | 2 | 2 | 2 | 2 | 0 | 2 | 1 | 2 | 0 | 1 | 70.00% |
| PTLS | (40) | Fernandez et al. | 2019 | 2 | 2 | 2 | 2 | 2 | 1 | 0 | 2 |  |  | 81.25% |
| PTLS | (41) | Shuib et al. | 2017 | 2 | 2 | 2 | 2 | 0 | 0 | 1 | 2 |  |  | 68.75% |
| PTLS | (42) | Yuan et al. | 2015 | 2 | 2 | 2 | 2 | 0 | 2 | 1 | 2 | 0 | 1 | 70.00% |
| PTLS | (43) | Alaimo et al. | 2015 | 2 | 2 | 2 | 2 | 1 | 0 | 1 | 2 |  |  | 75.00% |
| PTLS | (44) | Magoulas et al. | 2014 | 2 | 2 | 2 | 0 | 0 | 2 | 2 | 2 | 0 | 0 | 60.00% |
| PTLS | (45) | Lee et al. | 2013 | 2 | 2 | 2 | 2 | 0 | 0 | 1 | 2 |  |  | 68.75% |
| PTLS | (22) | Lee et al. | 2012 | 2 | 2 | 2 | 1 | 1 | 2 | 2 | 1 | 0 | 1 | 70.00% |
| PTLS | (46) | Goh et al. | 2012 | 2 | 2 | 2 | 1 | 1 | 2 | 2 | 1 | 0 | 2 | 75.00% |
| PTLS | (47) | Sanchez et al. | 2011 | 2 | 2 | 2 | 2 | 1 | 1 | 0 | 2 |  |  | 75.00% |
| PTLS | (48) | Zhang et al. | 2010 | 2 | 2 | 2 | 1 | 2 | 2 | 2 | 1 | 0 | 2 | 80.00% |
| PTLS | (49) | Nakamine et al. | 2008 | 2 | 2 | 2 | 2 | 1 | 0 | 0 | 2 |  |  | 68.75% |
| PTLS | (50) | Doco–Fenzy et al. | 2008 | 2 | 2 | 2 | 2 | 0 | 0 | 0 | 2 |  |  | 62.50% |
| PTLS | (51) | Potocki et al. | 2007 | 2 | 2 | 2 | 2 | 2 | 2 | 2 | 1 | 0 | 2 | 85.00% |
| SCA | (52) | Wang et al. | 2019 | 2 | 2 | 2 | 2 | 1 | 1 | 2 | 2 |  |  | 87.50% |
| SCA | (53) | Monte et al. | 2017 | 2 | 2 | 2 | 2 | 0 | 0 | 2 | 2 |  |  | 75.00% |
| SCA | (54) | Pereira et al. | 2015 | 2 | 2 | 2 | 1 | 0 | 0 | 1 | 2 |  |  | 62.50% |
| SCA | (55) | Chattopadhyay et al. | 2003 | 2 | 2 | 2 | 2 | 0 | 0 | 1 | 2 |  |  | 68.75% |
| SCA | (56) | Hayes et al. | 2000 | 2 | 2 | 2 | 1 | 1 | 0 | 1 | 2 |  |  | 68.75% |
| ASD | (57) | Trost et al. | 2022 | 2 | 2 | 2 | 2 | 2 | 2 | 2 | 2 |  |  | 100.00% |
| ASD | (58) | Mullegama et al. | 2015 | 2 | 2 | 2 | 2 | 1 | 1 | 2 | 2 |  |  | 87.50% |
| ASD | (59) | Redin et al. | 2014 | 2 | 2 | 2 | 2 | 0 | 0 | 1 | 2 |  |  | 68.75% |
| ASD | (60) | van et al. | 2009 | 2 | 2 | 2 | 1 | 0 | 0 | 1 | 2 |  |  | 62.50% |
| Schizophrenia | (61) | Haybaeck et al. | 2015 | 2 | 2 | 2 | 2 | 1 | 0 | 2 | 2 |  |  | 81.25% |
| Bipolar disorder | (61) | Haybaeck et al. | 2015 | 2 | 2 | 2 | 2 | 1 | 0 | 2 | 2 |  |  | 81.25% |
| Major depression | (61) | Haybaeck et al. | 2015 | 2 | 2 | 2 | 2 | 1 | 0 | 2 | 2 |  |  | 81.25% |

The Joanna Briggs Institute Critical Appraisal Checklist (2) for case reports, case series or analytical cross-sectional studies was used as appropriate for all included clinical studies. The full criteria checklists can be found online at <https://jbi.global/critical-appraisal-tools>. Scores: 2=the study/manuscript fulfilled the criteria; 1=it was unclear if the study/manuscript fulfilled the criteria; 0=the manuscript/study did not fulfil the criteria. The total score (%) is the sum score across all criteria.

**Reference**

1. Page MJ, McKenzie JE, Bossuyt PM, Boutron I, Hoffmann TC, Mulrow CD, et al. The PRISMA 2020 statement: an updated guideline for reporting systematic reviews. BMJ. 2021;372:n71.

2. Institute. TJB. Joanna Briggs Institute Reviewers' Manual: 2016 edition. The Joanna Briggs Institute, Adelaide, Australia. 2016.

3. Stern T, Hussein Y, Cordeiro D, Sadis H, Garin-Shkolnik T, Spiegel R, et al. Case Report: A Case of a Patient with Smith-Magenis Syndrome and Early-Onset Parkinson's Disease. Int J Mol Sci. 2024;25(15).

4. Cuk M, Unal B, Jandric N, Hayes CP, Walker M, Abraamyan F, et al. Novel RAI1:c.2736delC Variant in Smith-Magenis Syndrome: Identification by Whole Genome Sequencing and Joint Analysis. J Pers Med. 2024;14(9).

5. Wu X, Zhang L, Chen S, Li Y. A case of Smith-Magenis syndrome with skin manifestations caused by a novel locus mutation in the RAI1 gene. J Int Med Res. 2023;51(9):3000605231190553.

6. Yu R, Liu L, Chen C, Lin ZJ, Xu JM, Fan LL. A de novo mutation (p.S1419F) of Retinoic acid induced 1 is responsible for a patient with Smith-Magenis syndrome exhibiting schizophrenia. Gene. 2023;851:147028.

7. Sironi A, Bestetti I, Masciadri M, Tumiatti F, Crippa M, Pantaleoni C, et al. A unique Smith-Magenis patient with a de novo intragenic deletion on the maternally inherited overexpressed RAI1 allele. Eur J Hum Genet. 2022;30(11):1233-8.

8. Onesimo R, Delogu AB, Blandino R, Leoni C, Rosati J, Zollino M, et al. Smith Magenis syndrome: First case of congenital heart defect in a patient with Rai1 mutation. Am J Med Genet A. 2022;188(7):2184-6.

9. Kuroda Y, Ritter A, Mullegama SV, Izumi K. Mosaic RAI1 variant in a Smith-Magenis syndrome patient with total anomalous pulmonary venous return. Am J Med Genet A. 2022;188(10):3130-4.

10. Akkus N, Kilic B, Cubuk PO. Smith-Magenis Syndrome: Clues in the Clinic. J Pediatr Genet. 2020;9(4):279-84.

11. Abad C, Cook MM, Cao L, Jones JR, Rao NR, Dukes-Rimsky L, et al. A Rare De Novo RAI1 Gene Mutation Affecting BDNF-Enhancer-Driven Transcription Activity Associated with Autism and Atypical Smith-Magenis Syndrome Presentation. Biology (Basel). 2018;7(2).

12. Acquaviva F, Sana ME, Della Monica M, Pinelli M, Postorivo D, Fontana P, et al. First evidence of Smith-Magenis syndrome in mother and daughter due to a novel RAI mutation. Am J Med Genet A. 2017;173(1):231-8.

13. Yuan B, Neira J, Gu S, Harel T, Liu P, Briceño I, et al. Nonrecurrent PMP22-RAI1 contiguous gene deletions arise from replication-based mechanisms and result in Smith-Magenis syndrome with evident peripheral neuropathy. Hum Genet. 2016;135(10):1161-74.

14. Yeetong P, Vilboux T, Ciccone C, Boulier K, Schnur RE, Gahl WA, et al. Delayed diagnosis in a house of correction: Smith-Magenis syndrome due to a de novo nonsense RAI1 variant. Am J Med Genet A. 2016;170(9):2383-8.

15. Nijim Y, Adawi A, Bisharat B, Bowirrat A. First Case Report of Smith-Magenis Syndrome (SMS) Among the Arab Community in Nazareth: View and Overview. Medicine (Baltimore). 2016;95(3):e2362.

16. Thaker VV, Esteves KM, Towne MC, Brownstein CA, James PM, Crowley L, et al. Whole exome sequencing identifies RAI1 mutation in a morbidly obese child diagnosed with ROHHAD syndrome. J Clin Endocrinol Metab. 2015;100(5):1723-30.

17. Li Z, Shen J, Liang J, Sheng L. Congenital scoliosis in Smith-Magenis syndrome: a case report and review of the literature. Medicine (Baltimore). 2015;94(17):e705.

18. Dubourg C, Bonnet-Brilhault F, Toutain A, Mignot C, Jacquette A, Dieux A, et al. Identification of Nine New RAI1-Truncating Mutations in Smith-Magenis Syndrome Patients without 17p11.2 Deletions. Mol Syndromol. 2014;5(2):57-64.

19. Capra V, Biancheri R, Morana G, Striano P, Novara F, Ferrero GB, et al. Periventricular nodular heterotopia in Smith-Magenis syndrome. Am J Med Genet A. 2014;164a(12):3142-7.

20. Adams DR, Yuan H, Holyoak T, Arajs KH, Hakimi P, Markello TC, et al. Three rare diseases in one Sib pair: RAI1, PCK1, GRIN2B mutations associated with Smith-Magenis Syndrome, cytosolic PEPCK deficiency and NMDA receptor glutamate insensitivity. Mol Genet Metab. 2014;113(3):161-70.

21. Vieira GH, Rodriguez JD, Carmona-Mora P, Cao L, Gamba BF, Carvalho DR, et al. Detection of classical 17p11.2 deletions, an atypical deletion and RAI1 alterations in patients with features suggestive of Smith-Magenis syndrome. Eur J Hum Genet. 2012;20(2):148-54.

22. Lee CG, Park SJ, Yun JN, Yim SY, Sohn YB. Reciprocal deletion and duplication of 17p11.2-11.2: Korean patients with Smith-Magenis syndrome and Potocki-Lupski syndrome. J Korean Med Sci. 2012;27(12):1586-90.

23. Huang C, Yang YF, Zhang H, Xie L, Chen JL, Wang J, et al. Microdeletion on 17p11.2 in a Smith-Magenis syndrome patient with mental retardation and congenital heart defect: first report from China. Genet Mol Res. 2012;11(3):2321-7.

24. Vilboux T, Ciccone C, Blancato JK, Cox GF, Deshpande C, Introne WJ, et al. Molecular analysis of the Retinoic Acid Induced 1 gene (RAI1) in patients with suspected Smith-Magenis syndrome without the 17p11.2 deletion. PLoS One. 2011;6(8):e22861.

25. Sanford EF, Bermudez-Wagner K, Jeng LJ, Rauen KA, Slavotinek AM. Congenital diaphragmatic hernia in Smith-Magenis syndrome: a possible locus at chromosome 17p11.2. Am J Med Genet A. 2011;155a(11):2816-20.

26. Gamba BF, Vieira GH, Souza DH, Monteiro FF, Lorenzini JJ, Carvalho DR, et al. Smith-Magenis syndrome: clinical evaluation in seven Brazilian patients. Genet Mol Res. 2011;10(4):2664-70.

27. Truong HT, Dudding T, Blanchard CL, Elsea SH. Frameshift mutation hotspot identified in Smith-Magenis syndrome: case report and review of literature. BMC Med Genet. 2010;11:142.

28. Girirajan S, Mendoza-Londono R, Vlangos CN, Dupuis L, Nowak NJ, Bunyan DJ, et al. Smith-Magenis syndrome and Moyamoya disease in a patient with del(17)(p11.2p13.1). Am J Med Genet A. 2007;143a(9):999-1008.

29. Bi W, Saifi GM, Girirajan S, Shi X, Szomju B, Firth H, et al. RAI1 point mutations, CAG repeat variation, and SNP analysis in non-deletion Smith-Magenis syndrome. Am J Med Genet A. 2006;140(22):2454-63.

30. Vlangos CN, Wilson M, Blancato J, Smith AC, Elsea SH. Diagnostic FISH probes for del(17)(p11.2p11.2) associated with Smith-Magenis syndrome should contain the RAI1 gene. Am J Med Genet A. 2005;132a(3):278-82.

31. Schoumans J, Staaf J, Jönsson G, Rantala J, Zimmer KS, Borg A, et al. Detection and delineation of an unusual 17p11.2 deletion by array-CGH and refinement of the Smith-Magenis syndrome minimum deletion to approximately 650 kb. Eur J Med Genet. 2005;48(3):290-300.

32. Girirajan S, Elsas LJ, 2nd, Devriendt K, Elsea SH. RAI1 variations in Smith-Magenis syndrome patients without 17p11.2 deletions. J Med Genet. 2005;42(11):820-8.

33. Spadoni E, Colapietro P, Bozzola M, Marseglia GL, Repossi L, Danesino C, et al. Smith-Magenis syndrome and growth hormone deficiency. Eur J Pediatr. 2004;163(7):353-8.

34. Myers SM, Challman TD. Congenital heart defects associated with Smith-Magenis syndrome: two cases of total anomalous pulmonary venous return. Am J Med Genet A. 2004;131(1):99-100.

35. Bi W, Saifi GM, Shaw CJ, Walz K, Fonseca P, Wilson M, et al. Mutations of RAI1, a PHD-containing protein, in nondeletion patients with Smith-Magenis syndrome. Hum Genet. 2004;115(6):515-24.

36. Slager RE, Newton TL, Vlangos CN, Finucane B, Elsea SH. Mutations in RAI1 associated with Smith-Magenis syndrome. Nat Genet. 2003;33(4):466-8.

37. Deginet E, Abdissa D, Hailu T. Potocki-Lupski Syndrome in Ethiopian Child: A Case Report. Pediatric Health Med Ther. 2024;15:129-31.

38. Grama A, Sîrbe C, Miclea D, Cǎinap SS, Huniadi D, Bulata B, et al. Case Report: Potocki-Lupski Syndrome in Five Siblings. Front Pediatr. 2021;9:698629.

39. Ciaccio C, Pantaleoni C, Milani D, Alfei E, Sciacca FL, Canafoglia L, et al. Neurological phenotype of Potocki-Lupski syndrome. Am J Med Genet A. 2020;182(10):2317-24.

40. Fernandez-Hernandez L, Navarro-Cobos MJ, Alcantara-Ortigoza MA, Ramos-Angeles SE, Molina-Alvarez B, Diaz-Cuellar S, et al. Report of a patient with a de novo non-recurrent duplication of 17p11.2p12 and Yq11 deletion. Molecular Cytogenetics. 2019;12(1):35.

41. Shuib S, Saaid NN, Zakaria Z, Ismail J, Abdul Latiff Z. Duplication 17p11.2 (Potocki-Lupski Syndrome) in a child with developmental delay. Malays J Pathol. 2017;39(1):77-81.

42. Yuan B, Harel T, Gu S, Liu P, Burglen L, Chantot-Bastaraud S, et al. Nonrecurrent 17p11.2p12 Rearrangement Events that Result in Two Concomitant Genomic Disorders: The PMP22-RAI1 Contiguous Gene Duplication Syndrome. Am J Hum Genet. 2015;97(5):691-707.

43. Alaimo JT, Mullegama SV, Thomas MA, Elsea SH. Copy number loss upstream of RAI1 uncovers gene expression regulatory region that may impact Potocki-Lupski syndrome diagnosis. Mol Cytogenet. 2015;8:75.

44. Magoulas PL, Liu P, Gelowani V, Soler-Alfonso C, Kivuva EC, Lupski JR, et al. Inherited dup(17)(p11.2p11.2): expanding the phenotype of the Potocki-Lupski syndrome. Am J Med Genet A. 2014;164a(2):500-4.

45. Lee CG, Park SJ, Yim SY, Sohn YB. Clinical and cytogenetic features of a Potocki-Lupski syndrome with the shortest 0.25Mb microduplication in 17p11.2 including RAI1. Brain Dev. 2013;35(7):681-5.

46. Goh ESY, Perez IC, Canales CP, Ruiz P, Agatep R, Yoon G, et al. Definition of a critical genetic interval related to kidney abnormalities in the Potocki-Lupski syndrome. American Journal of Medical Genetics, Part A. 2012;158 A(7):1579-88.

47. Sanchez-Valle A, Pierpont ME, Potocki L. The severe end of the spectrum: Hypoplastic left heart in Potocki-Lupski syndrome. Am J Med Genet A. 2011;155a(2):363-6.

48. Zhang F, Potocki L, Sampson JB, Liu P, Sanchez-Valle A, Robbins-Furman P, et al. Identification of uncommon recurrent Potocki-Lupski syndrome-associated duplications and the distribution of rearrangement types and mechanisms in PTLS. Am J Hum Genet. 2010;86(3):462-70.

49. Nakamine A, Ouchanov L, Jiménez P, Manghi ER, Esquivel M, Monge S, et al. Duplication of 17(p11.2p11.2) in a male child with autism and severe language delay. Am J Med Genet A. 2008;146a(5):636-43.

50. Doco-Fenzy M, Holder-Espinasse M, Bieth E, Magdelaine C, Vincent MC, Khoury M, et al. The clinical spectrum associated with a chromosome 17 short arm proximal duplication (dup 17p11.2) in three patients. Am J Med Genet A. 2008;146A(7):917-24.

51. Potocki L, Bi W, Treadwell-Deering D, Carvalho CM, Eifert A, Friedman EM, et al. Characterization of Potocki-Lupski syndrome (dup(17)(p11.2p11.2)) and delineation of a dosage-sensitive critical interval that can convey an autism phenotype. Am J Hum Genet. 2007;80(4):633-49.

52. Wang P, Chen Z, Peng Y, Cao L, Li X, Wang C, et al. (CAG)(n) loci as genetic modifiers of age at onset in patients with spinocerebellar ataxia type 1 from mainland China. Eur J Neurol. 2019;26(8):1130-6.

53. Monte TL, Pereira FS, Reckziegel EDR, Augustin MC, Locks-Coelho LD, Santos ASP, et al. Neurological phenotypes in spinocerebellar ataxia type 2: Role of mitochondrial polymorphism A10398G and other risk factors. Parkinsonism Relat Disord. 2017;42:54-60.

54. Pereira FS, Monte TL, Locks-Coelho LD, Silva AS, Barsottini O, Pedroso JL, et al. ATXN3, ATXN7, CACNA1A, and RAI1 Genes and Mitochondrial Polymorphism A10398G Did Not Modify Age at Onset in Spinocerebellar Ataxia Type 2 Patients from South America. Cerebellum. 2015;14(6):728-30.

55. Chattopadhyay B, Ghosh S, Gangopadhyay PK, Das SK, Roy T, Sinha KK, et al. Modulation of age at onset in Huntington's disease and spinocerebellar ataxia type 2 patients originated from eastern India. Neurosci Lett. 2003;345(2):93-6.

56. Hayes S, Turecki G, Brisebois K, Lopes-Cendes I, Gaspar C, Riess O, et al. CAG repeat length in RAI1 is associated with age at onset variability in spinocerebellar ataxia type 2 (SCA2). Hum Mol Genet. 2000;9(12):1753-8.

57. Trost B, Thiruvahindrapuram B, Chan AJS, Engchuan W, Higginbotham EJ, Howe JL, et al. Genomic architecture of autism from comprehensive whole-genome sequence annotation. Cell. 2022;185(23):4409-27 e18.

58. Mullegama SV, Alaimo JT, Chen L, Elsea SH. Phenotypic and molecular convergence of 2q23.1 deletion syndrome with other neurodevelopmental syndromes associated with autism spectrum disorder. Int J Mol Sci. 2015;16(4):7627-43.

59. Redin C, Gérard B, Lauer J, Herenger Y, Muller J, Quartier A, et al. Efficient strategy for the molecular diagnosis of intellectual disability using targeted high-throughput sequencing. J Med Genet. 2014;51(11):724-36.

60. van der Zwaag B, Franke L, Poot M, Hochstenbach R, Spierenburg HA, Vorstman JA, et al. Gene-network analysis identifies susceptibility genes related to glycobiology in autism. PLoS One. 2009;4(5):e5324.

61. Haybaeck J, Postruznik M, Miller CL, Dulay JR, Llenos IC, Weis S. Increased expression of retinoic acid-induced gene 1 in the dorsolateral prefrontal cortex in schizophrenia, bipolar disorder, and major depression. Neuropsychiatr Dis Treat. 2015;11:279-89.
